# Falls Sensei: a serious 3D exploration game to enable the detection of extrinsic home fall hazards for older adults

**DOI:** 10.1186/s12911-019-0808-x

**Published:** 2019-04-16

**Authors:** Arthur G. Money, Anita Atwal, Emily Boyce, Sophie Gaber, Susan Windeatt, Kyriakos Alexandrou

**Affiliations:** 1 Department of Computer Science, Brunel University London, Uxbridge, UB8 3PH UK; 2 Faculty of Health and Social Care, London South Bank University, London, SE1 0AA UK; 3 North London Forensic Service, Barnet, Enfield and Haringey Mental Health NHS Trust, Enfield, EN2 8JL UK; 4 Department of Neurobiology, Care Sciences & Society, Division of Occupational Therapy, Karolinska Institutet, SE-171 77 Stockholm, Sweden; 5 CIS Westminster Rehabilitation Service, Gordon Hospital London, CNWL NHS Foundation Trust, SW1V 2RH, London, UK; 6 Programonks, Eleftheriou Venizelou 1 Athienou, 7600 Larnaca, Cyprus

**Keywords:** Serious games, 3D, Game-based learning, Health education, Falls prevention, Older adults, Occupational therapy, Virtual reality

## Abstract

**Background:**

Falls are the main cause of death and injury for older adults in the UK. Many of these falls occur within the home as a result of extrinsic falls risk factors such as poor lighting, loose/uneven flooring, and clutter. Falls education plays an important role in self-management education about extrinsic hazards and is typically delivered via information leaflets, falls apps, and educational booklets. Serious games have the potential of delivering an engaging and informative alternative to traditional methods but almost exclusively, these are currently delivered as exergaming applications that focus solely on intrinsic falls risk factors. This study presents ‘Falls Sensei’ a first-person 3D exploration game that aims to educate older adults about extrinsic falls risk factors within the home environment. After presenting Falls Sensei, game usability and older adults’ perceptions and attitudes towards using the game in practice are explored.

**Methods:**

This study involved 15 community dwelling older adults. After playing the Falls Sensei game, participants completed a Systems Usability Scale (SUS) questionnaire and post task interview, and follow-up interviews three weeks later. Inductive and deductive thematic template analysis, informed by the Unified Theory of Acceptance and Use of Technology model, was used to analyse the think-aloud, post-task and follow-up interview transcripts. Descriptive statistical analysis and one-sampled t-tests were used to analyse log-file data and SUS responses.

**Results:**

Three high-level themes emerged from the analysis of transcriptions: Performance Expectancy; Effort Expectancy; Social Influence. The SUS score was 77.5/100 which indicates ‘Good’ levels of usability. Interestingly, reported usability of the game increased with participant age. Participants were positive about the usability of the game (*p* < = 0.05 for 9/10 items). The most memorable fall hazards were those most commonly encountered in the game or those most challenging to participants.

**Conclusions:**

The results support the use of serious games as an engaging tool for educating older adults about extrinsic falls risk factors. Awareness of home hazard detection was raised by the game, and some older adults became more aware for the need to adapt their own homes after gameplay. Further research would be needed to draw comparisons with established interventions.

## Background

Falls are the leading cause of death by injury for older adults in the UK [1]. The chance of falling is comparatively low for those aged 50 and under but raises by over 50% for adults over eighty [1]. Falls have a serious consequence not only for the person in relation to the healthy life years lost due to disability, but in addition to this there is a psychological impact and a fear of future falling that can lead to inactivity, loss of confidence and societal withdrawal [2, 3]. There are also considerable associated issues for health and social care providers, since social care costs are estimated to be almost 40% higher in the twelve months following a fall [4]. The World Health Organisation (WHO) suggests age-friendly living environments must address multiple home-related factors to enable older adults to feel comfortable and safe [5]. Data collected from 116 USA households during the checklist evaluation [6] found the three most common fall hazards detected were found in the bathroom (absence of: a non-slip bath/shower mat, grab bars, a non-slip bathroom rug). Poor lighting was reported in 11% of households. Other fall hazards found included loose rugs or carpeting, clutter and uneven flooring. Relatively easy changes can be made that do not require the intervention of a health care professional but require knowledge and understanding of falls. Technology may advance this further. For example, a relatively new innovation includes a robot control system for in-home environment screening of falls hazards [7]. This system enables a health professional to assess a house remotely and to interact with the user using telepresence.

Falls education strategies are increasingly being used to educate persons about falls hazards within the home. Education is an important aspect of self-management and may include mediums such as mobile phone falls applications [8] and or educational booklets [6]. An alternative medium could be extending the use of serious games within health and social care. Serious games can be defined as games that have been designed to have an explicit educational purpose, and are not intended to be played primarily for entertainment, but may still be entertaining to play [9]. Serious games have been developed for health promotion purposes and used within education establishments [10, 11], for example within the airline industry to educate passengers about patient safety [12], to discuss sensitive topics such as challenging sexual relationships, and mental health and wellbeing related issues [13, 14]. Games used for falls prevention have almost exclusively concentrated on improving health through exercise known as “exergaming”, which is defined as a form of serious gaming that requires body movement to make progress in the game, thereby increasing levels of physical exercise [15]. This is not surprizing since balance training and exercise programmes are recommended as fall preventative interventions [16]. Meta-analyses including large Cochrane reviews comparing exercise interventions with placebo or non-exercise programmes have found significant reductions in falls risk, actual falls, fracture risk and falls requiring medical attention [17, 18]. These games use either standard or customised physical apparatus attached to a games console or computer the most common of which used is the Nintendo Wii. One study found that such games are not more effective than traditional exercise programmes [19] they have been found to be equally effective and have the advantage of delivering entertainment as an additional intentional motivation [20]. It is important to ascertain whether serious games have a role to play in promoting falls safety.

This study presents “Falls Sensei” a serious game which was developed with the aim of educating older adults on home environmental fall hazards in an engaging fashion. The game consists of a first-person style walk-through of a home environment with four levels (Kitchen, Bathroom, Bedroom, Lounge and Stairs). Players are challenged to find twenty-six hazards, some being repeated for emphasis, and hints to aid hazard detection are displayed at regular intervals. An initial pilot study with ten participants suggested potential for this approach in fall hazard education for older adults. The aims of this research paper are threefold:

1. To present Falls Sensei, a serious 3D exploration game to increase awareness of environmental fall hazards (extrinsic) that are apparent within the home.
2. To evaluate the overall game usability from an older adult perspective.
3. To explore older adults’ perceptions of using Falls Sensei, the factors that would affect the adoption of this application, and the extent to which modification of falls prevention related behaviour can occur as a consequence of playing the Falls Sensei game.

## The falls sensei game

### System architecture and game logic

**Fig. 1:**
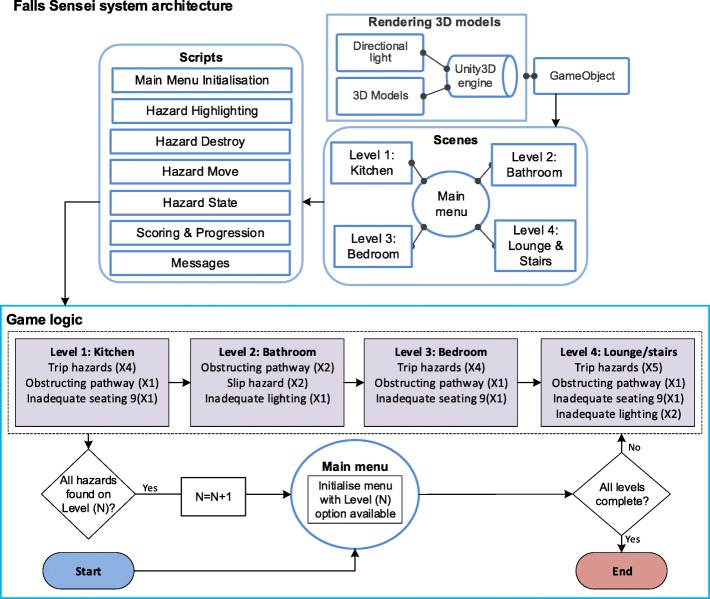
Falls Sensei system architecture and game logic

Falls Sensei is a first-person 3D exploration game, with four ‘levels’ which correspond with four key living areas within the home: Kitchen, Bathroom, Bedroom, Lounge and stairs. The application was developed in Unity3D and uses a Unity3D engine to generate a GameObject which contains a suite of 3D Models and associated Scenes which are presented at each respective game level. Several custom scripts drive the game narrative and logic. The Main Menu Initialisation script presents the Main Menu to the player. The Hazard Highlighting script monitors the in-game navigation of the player and highlights potential hazardous items ‘OnMouseOver’ within each respective scene. On correct selection of a fall hazard, Hazard Destroy removes the hazard from the scene and Hazard Move either removes the hazard from the scene or moves the hazardous item to a non-hazardous location. Hazard State monitors the number of hazards that have been successfully identified and triggers messages via the Messages script to either help the player identify unfound hazards or to positively enforce the successful identification of a hazard. The Messages script also communicates additional educational content about a hazard when it is successfully found, or conversely, when an item is incorrectly selected as a falls hazard a justification for its exclusion as a hazard is provided. The Scoring and Progression script keeps a running total of the score and monitors the overall progress within a scene and in the game overall. Figure 1 presents the high-level Falls Sensei system architecture and an overview of the basic level progression logic.

### Game walkthrough

**Fig. 2:**
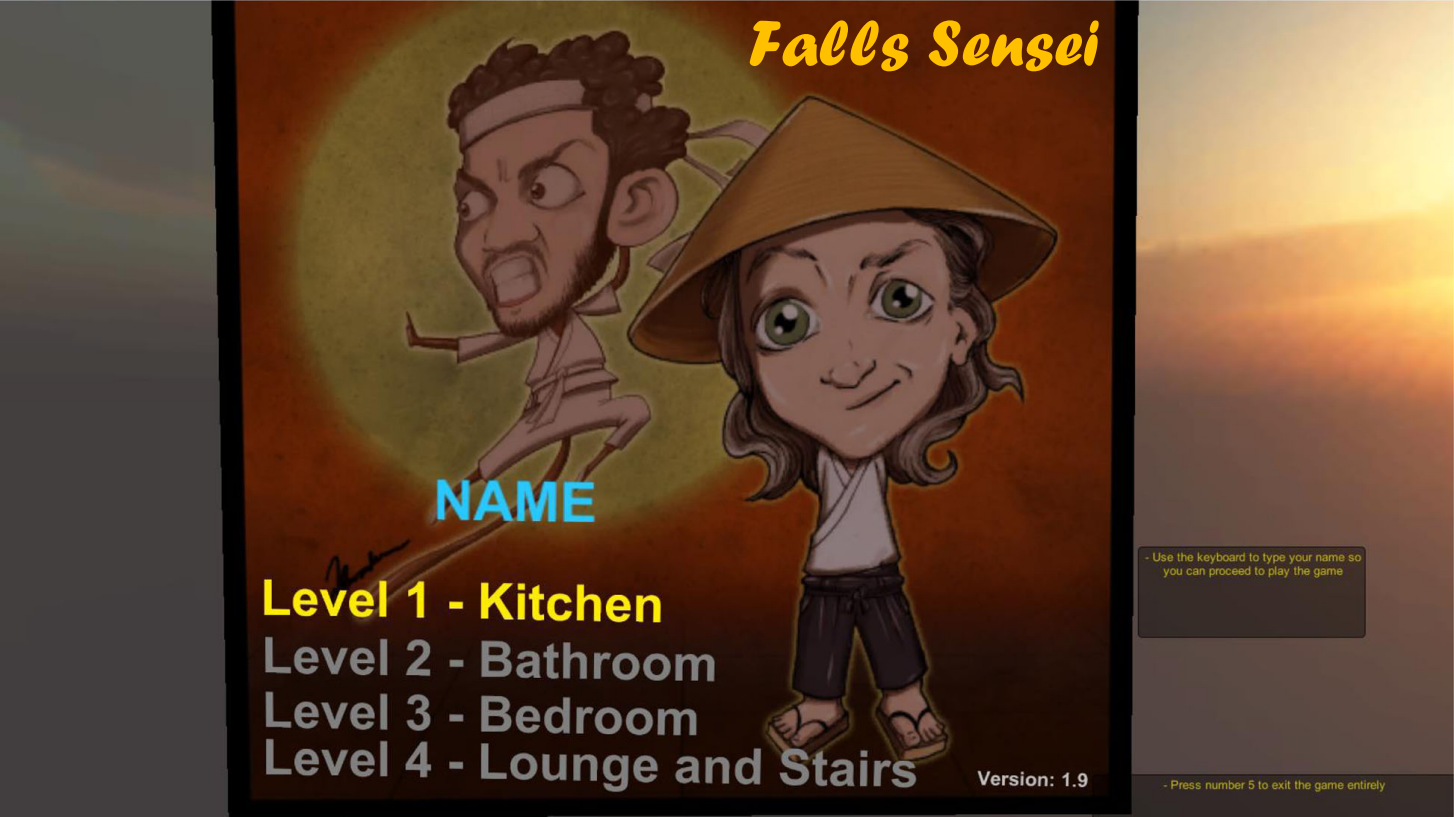
Falls Sensei main menu

On opening the application, the user is presented with the main menu screen, displaying the four levels and prompting them to type in a name. The main menu screen is presented in Fig. 2.

**Fig. 3:**
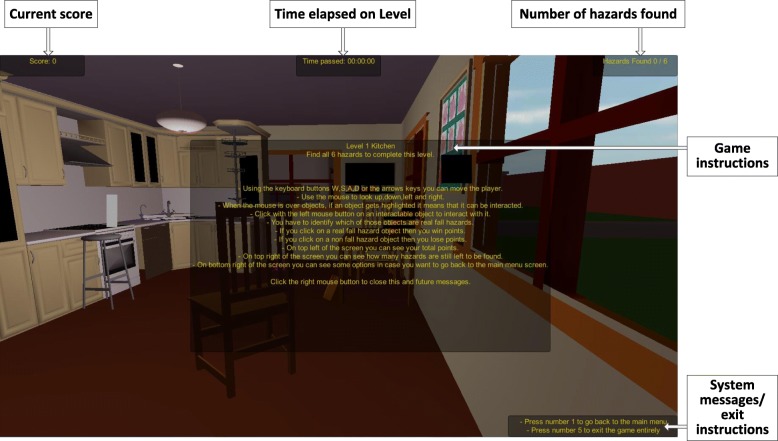
Level 1 - Kitchen area and initial game instructions

Each level displays a modelled living area that, by default, incorporates between five and nine fall hazards into the scene. The challenge for the user is to navigate/explore each of the four living areas within the home whilst searching for hazards. The game deploys a sequential progression mechanism, which allows the player to progress through the four levels of the game in sequential order, starting at Level 1 – Kitchen, and finishing at Level 4- Lounge and Stairs. The user is required to find all hazards at a given level before being permitted to progress to the next level i.e. opening the room door and gaining access to the next level and associated living area. Common fall hazards that are presented in the home include: objects obstructing pathways, trip hazards, inadequate lighting, inappropriate seating, reach hazards. Figure 3 presents the Level 1 Kitchen scene along with initial game instructions that the user sees on entering the scene for the first time.

**Fig. 4:**
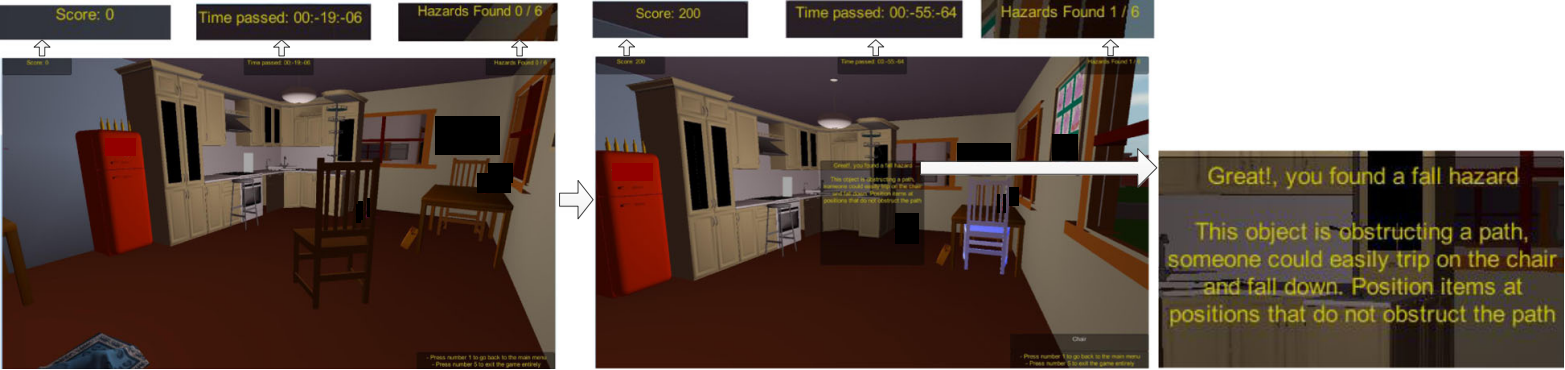
**a** Chair obstructing path, (**b**) Chair identified and repositioned in a non-hazard (**c**) Expanded on-screen game notification to user

On correct identification of a fall hazard, further information about the identified fall hazard is presented on-screen and the hazard is automatically rectified in the game environment so that the player can see an example of the item positioned in a non-hazardous location. The user is awarded points for each hazard they identify correctly. Figure 4a shows the Kitchen scene with a chair obstructing a path, (B) shows the chair repositioned in a safe place repositioned under the kitchen table, and (C) presents the on-screen notification that the system displays to the user on selecting the chair fall hazard correctly.

**Fig. 5:**
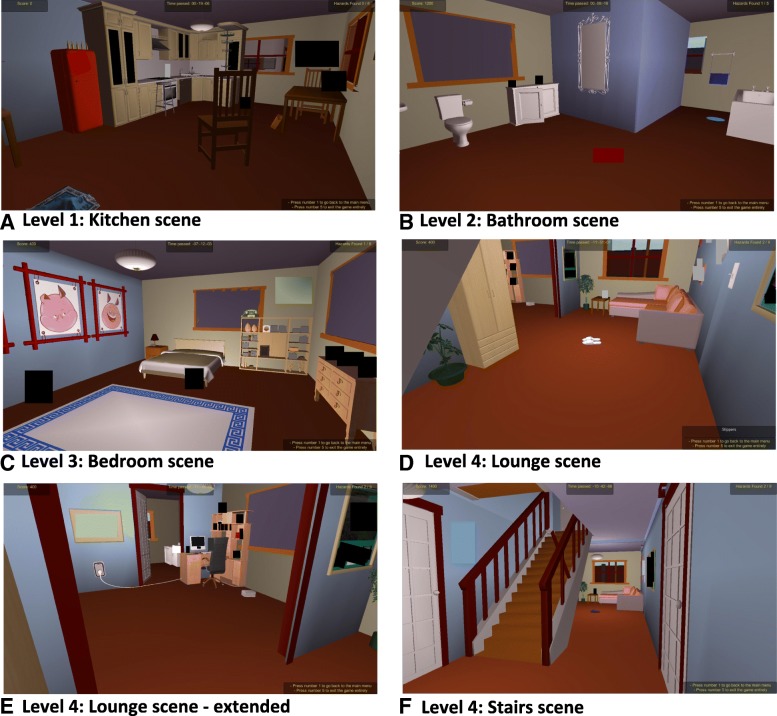
Example scenes from all four game levels

Also of note that the score is zero in Fig. 4a (“Score: 0”) prior to successful selection of the chair fall hazard with a total of “Hazards Found: 0/6” in screen (A), which is then updated to “Score: 200” (200 points per hazard found are awarded to the user), along with “Hazards Found: 1/6” on successful identification of a fall hazard. The system also positively reinforces the successful identification of a fall hazard by congratulating the user for finding a hazard successfully and explains why the chair was considered to be a hazard in its initial position via an on-screen text message. On-screen messages are all tailored to the range of objects and respective fall hazards they represent throughout the game. A similar protocol is followed throughout all levels of the game. Figure 5 presents example scenes from the four game levels.

## Methods

A mixed methods data collection and analysis protocol was used to address the specific research aims of this study, details of which are presented in this section.

### Participants

Fifteen participants were recruited initially from adults attending an Active 50s gym group on a university campus. Nine identified themselves as female and six as male. The participant’s age ranged between 50 and 80 years old. One participant declined to give their age. Six participants were aged 50–60 and 60–70, seven aged between 70 and 80. The inclusion criteria were age (fifty or over, basic computer knowledge and clear vision with or without correction (glasses/contact lenses) due to the game currently not being size adjustable. Previous research has suggested that if participants do not have a basic level of computer literacy (for example being able to send and receive email) they became too focused on hardware issues and were not able to offer constructive criticism of the software being studied [21]. Due to the beta functionality of the developed game software it was necessary to exclude participants with visual impairments. This was because the game, and more crucially the in-game instructional text, was not easily size adjustable for low vision. All participants were able to use the mouse and keyboard successfully although one required an adjustment to left-handed mouse control. The desktop nature of the game software, with regard to key pressing and mouse navigational control, did exclude those with upper limb functional impairments. Table 1 presents an overview of participant demographics.

**Table 1:** Participant demographics

Pseudonym	Age Bracket	Gender
Laura	66–70	Female
Daniel	76–80	Male
Matthew	71–75	Male
Joshua	71–75	Male
Aimee	71–75	Female
Lucy	61–65	Female
Ethan	66–70	Male
Thomas	71–75	Male
Charlotte	71–75	Female
Deborah	50–55	Female
Christopher	Not disclosed	Male
Karen	66–70	Female
Kesia	76–80	Female
Kate	50–55	Female
Emily	56–60	Female

### Protocol and instrumentation

Falls Sensei interactive usability sessions were held with all participants. Each participant was invited to attend a one-to-one session held at a London University. All sessions had a researcher present and written consent was obtained from all participants. On arrival at the session, any questions participants had, were answered. The think-aloud technique was then explained, and participants asked to think-aloud whilst playing the game with a view to ascertaining community dwelling older adults’ perceptions of the game as they played it. The think-aloud technique, commonly used to research technology acceptance usability [22, 23]. The technique requires participants to verbalise their thoughts and reasoning for actions either after or during task completion. It has a theoretical basis in Vygotsky’s theories of psychological learning development through internalised speech [24]. A large scale meta-analysis of 94 studies employing the technique shows this method of data collection has no effect on task performance, meaning the use of the technique will not affect the way participants play the game [25]. Participants were then provided with on-screen game instructions prior to playing the Falls Sensei game. On completion of the game, each participant was interviewed with a view to reflecting on their experience further. The Falls Sensei application also recorded a log-file of participant in-game performance which included overall duration, time spent on each game level, the fall hazards that were identified and the order in which they were identified. Participants were then asked to complete SUS questionnaire. The system usability scale (SUS) was administered after playing the game to ascertain user’s subjective satisfaction and acceptance of the game. SUS is comprised of 10 statements which users are required rate using a 5-point Likert type scale ranging from 1-strongly disagree to 5-strongly agree. Each SUS item was modified in accordance with the SUS practitioner guidelines, by replacing the word “system” with “Falls Sensei game” [26]. The Systems Usability Scale has the benefit of being a short and simple scale which has good validity and reliability [27]. This may be useful in determining how the game may be used in practice; for instance, whether users would need support in playing or may prefer to play at home. There are numerous more comprehensive game experience inventories that may have been used or measure constructs such as playability, enjoyment, engagement, flow in addition to more generic system usability criteria [28–30], however, these tend to be lengthy inventories and significantly more complex than the SUS. Taking into account the numerous tasks that older adult participants were required to carry out as part of this study, and to avoid cognitive stress and tester fatigue, it was felt that SUS was a more appropriate inventory to use in this context, given its simplicity, reliability and validity as a general usability measurement inventory. Consent was then confirmed for follow up telephone call scheduled to take place 3 weeks after playing game. Telephone interviews were conducted 3 weeks after the interactive usability session to further explore user perceptions of the game and to ascertain whether the game changed the behaviour the participants. Telephone interviews are a convenient means of collecting rich data although telephone interviews can lose valuable data without visual cues gathered from face-to-face interviews [31]. Once data collection was completed, all think-aloud sessions and interviews were transcribed verbatim.

### Data analysis

Deductive thematic template analysis was used to analyse the think aloud transcripts [32], where analysis is driven by a pre-defined template (a priori) of themes based on a theoretical framework [33]. The first stage involved creating a template which used three key determinants of technology use as defined by the Unified Theory of Acceptance and Use of Technology (UTAUT) Model [34, 35]. UTAUT is a widely used and empirically validated model of technology acceptance which integrates eight existing models and has been shown to account for 70% of user intentions to adopt and use new technologies. Hence the analysis considered the three key UTAUT determinants of intention to adopt new technology: Performance Expectancy (PE); Effort Expectancy (EE); Social Influence (SI). The entire corpus was perused and coded; identifying specific extracts from the data that related to the three UTAUT themes and other high-level emergent themes. The corpus was then perused iteratively through several stages of splicing, linking, deleting and reassigning sub-themes within the context of the high-level themes. Finally, a template covering the finalised themes and sub-themes was proposed. Overall SUS scores were calculated and interpreted according to the acceptability range, and the adjective and school grading scales [36]. This involved calculating a mean SUS representative value on a 100-point rating scale for each sample. These scores were then mapped to descriptive adjectives (Best imaginable, Excellent, Good, OK, Poor, Worst Imaginable), an acceptability range (Acceptable, Marginal-High, Marginal-Low, Not acceptable) and a school grading scale (i.e. 90–100 = A, 80–89 = B etc.)*. The baseline adjective and acceptability ranges are derived from* a sample of over 3000 software applications [37]. Additional statistical analysis was performed using one-sample t-test to establish whether there were significant differences between the respective mean SUS scores and the mid-point value of three (of the five-point Likert type scale responses) for each individual SUS item. Telephone interviews were analysed using inductive thematic analysis which followed a similar process to that of the think-aloud transcript analysis, but the high-level themes and sub-themes emerged purely from iterative thematic analysis of the dataset.

## Results

### Log-file findings

Analysis of the log-file data collected during trails revealed that the average time a participant spent actively playing the game from start to finish was 17 min and 56 s. The average time spent thinking about, and successfully identifying a hazard across all levels, was 42 s. Individual hazard thinking times were not recorded, however, each hazard and the order in which each hazard was successfully identified at each level was recorded. Table 2 presents a summary of the results of the log-file data analysis.

**Table 2:** Average gameplay hazard detection rankings & time taken per level.

Game Level	In-Game Hazards (in ranked order)	Avg. Identification Rank Order	Gap score	Df	Avg. Time to complete Level (mm:ss)	*P*-value (2-tail)Avg. Time to identify a Hazard per level (mm:ss)
		Rank	Mean	Modal		
1: Kitchen	Carpet / Rug	1	1.5	1	04:32 (*SD* = 02:04)	00:49 (*SD* = 00:21)
	Juice Carton	2	2.7	2		
	Dragon Toy	3	3.5	3,4		
	Dining Chair	4	3.7	3		
	Backless Stool	5	4.6	4,5,6		
	Wine Bottles	6	4.9	6		
2: Bathroom	Sneakers/Shoes	1	1.5	1	03:28 (*SD* = 01:33)	00:42 (*SD* = 00:19)^*^
	Open Cabinet Door	2	2.3	1,2		
	Water Puddle	3	2.7	3		
	Light (Switched off)	4	4	5		
	Bath Tub	5	4.3	4		
3: Bedroom	Human Figure	1	2	1,2	03:20 (*SD* = 01:40)	00:33 (*SD* = 00:17)
	Teddy Bear	2	2.1	1		
	Book	3	3.1	3		
	Puff Chair	4	3.1	5		
	Telephone	5	4.3	4		
	Light (Switch)	6	5.3	6		
4: Lounge & Stairs	Slippers	1	2.1	2	06:36 (*SD* = 03:00)	00:44 (*SD* = 00:20)
	Broken Stair	2	2.9	1		
	Books (on floor)	3	4.4	4,5		
	Light (Switch near	4	4.7	3		
	Books (on stairs)	5	5.3	4		
	Flex (across doorway	6	5.3	6		
	Light (Switch in hall)	7	5.5	3,8		
	Clothing Pile (on	8	6.7	6,7		
	Iron (on floor)	9	7.5	6,7		

The average time taken to identify a hazard at each level, was longest in duration (00:49) at Level 1: Kitchen. This may understandably be the case when considering that all participants must commence their play at Level 1, and hence there may be some time overhead incorporated into becoming accustomed to the gameplay environment, the keyboard controls, and getting to grips with the game rules and so forth. The average time to identify a hazard then steadily reduced at Level 2 (00:42, SD =), and then again at Level 3 (00:33), but then increased again at Level 4 (00:44). The increase in duration for level 4 may be explained by the fact that the Lounge & Stairs scene was considerably more complex that the previous three levels. In essence, there were three living areas to navigate at this level: the entrance hall which included stairs; the lounge area; and the study area which was.

located through an open archway off the lounge. Similarly, the average time taken to complete each level also followed this pattern. Level 1 one average took participants 04:32 to complete, reducing to 03:28 for Level 2, and 03:20 for Level 3, then increasing to 06:36 for Level 4. It should be noted, however, that the number of hazards in each respective level followed this order too, with six hazards presented in Level 1, reducing to five hazards at Level 2, increasing back to six hazards at Level 3 and then to nine hazards at Level 4. Therefore, reinforcing the observed pattern to some degree.

### SUS findings

One out of the 15 participants submitted incomplete SUS questionnaires, and hence these were removed from the sample. Therefore, a total of 14 SUS questionnaires were analysed. With regards to the overall SUS score reported by participants for the Falls Sensei game, the overall mean SUS score for the cohort was 77.50 out of 100, which according to the evaluation criteria for SUS [37], indicates that the application delivers ‘Good’ (Descriptive adjective), ‘High acceptable’ (Acceptability range), and Grade C (School grading scale) usability. This mean SUS score is above the mean SUS baseline score of 70 out of 100 which is the average score achieved by > 5000 software applications which provide the comparative SUS benchmark for comparison [37].

A follow-up statistical analysis of the individual SUS items against the mid-point of 3.00, was carried out to identify the extent to which individual SUS items were similar or significantly different from the mid-point. To conduct this analysis, the negative SUS items (S2, S4, S6, S8, and S10) were reversed so that scores above 3.00 indicated a positive response. Table 3 presents the results of this analysis.

**Table 3:** Mean SUS score and mid-point comparison

SUS item	Mid-point	Falls Sensei application	Gap score	Df	t-value	*P*-value (2-tail)
		Mean ± SD				
S1: I think that I would like to use this Falls Sensei game frequently.	3.00	3.07 ± 1.43	0.07	13	0.19	0.856
S2: I found the Falls Sensei game unnecessarily complex.^a^	3.00	4.36 ± 0.93	1.36	13	5.47	0.000^*^
S3: I thought the Falls Sensei game was easy to use.	3.00	3.92 ± 0.91	0.92	13	3.79	0.002^*^
S4: I think that I would need the support of a technical person to be able to use this Falls Sensei game ^a^	3.00	4.07 ± 1.07	1.07	13	3.74	0.002^*^
S5: I found the various functions in this Falls Sensei game were well integrated.	3.00	3.86 ± 0.74	0.86	13	3.71	0.003^*^
S6: I thought there was too much inconsistency in this Falls Sensei game.^a^	3.00	4.42 ± 1.09	1.42	13	4.91	0.000*
S7: I would imagine that most people would learn to use this Falls Sensei game very quickly.	3.00	4.36 ± 0.74	1.36	13	6.82	0.000^*^
S8: I found the Falls Sensei game very cumbersome to use.^a^	3.00	4.29 ± 1.07	1.29	13	4.50	0.001^*^
S9: I felt very confident using the Falls Sensei game	3.00	4.43 ± 0.65	1.42	13	8.27	0.000^*^
S10: I needed to learn a lot of things before I could get going with this Falls Sensei game.^a^	3.00	4.21 ± 0.89	1.21	13	5.09	0.000^*^

^a^Responses of negative items were reversed to align with positive items, higher scores indicate positive responses

*Indicates statitisically significant <= 0.05 confidence level

**Fig. 6:**
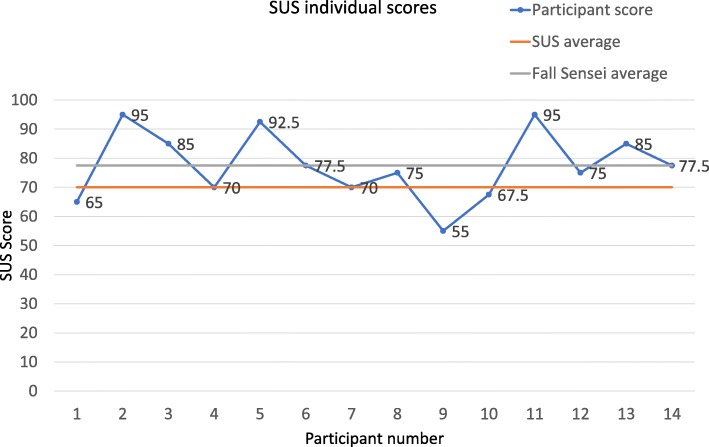
SUS individual scores, compared with SUS benchmark and Falls Sensei average

Mean scores for all 10 SUS items, in absolute terms, were above the neutral mid-point of 3.00, which indicates that participants tended to be positive about the Falls Sensei game in terms of the SUS items. Furthermore, in terms of statistical significance, mean responses to all nine out of 10 SUS items were significantly higher than the mid-point benchmark. To further consider overall SUS scores for each participant, Fig. 6 presents the individual SUS scores for each participant compared with the SUS benchmark (70/100), and the mean average achieved by Falls Sensei overall (77.50/100).

**Fig. 7:**
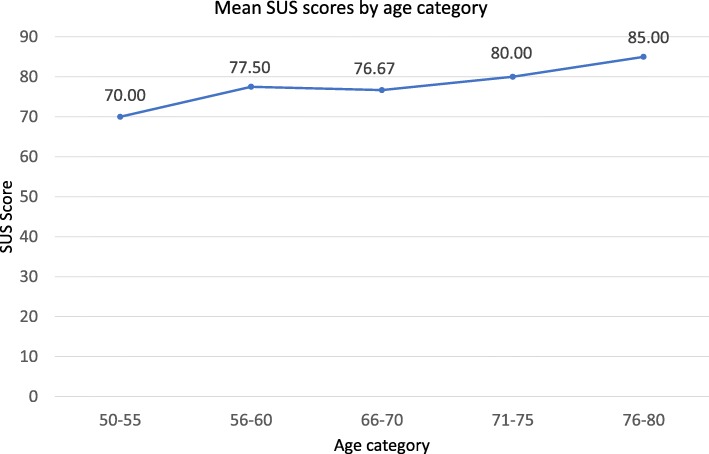
Mean SUS scores by age range

Eleven of the 14 participants who completed the SUS scored the game on or above the usability average of 70 out of 100. Three participants scored the game as below the SUS average for usability. The mean scores for participant age categories were also analysed. Usability and age scored means were therefore calculated. Figure 7 presents the mean SUS score per age category.

The results reveal that mean SUS usability scores increased as age category increases. The mean SUS score for participants aged 50–55 was 70 out of 100, this rose to 77.5 for participants aged 56–60, rising to 85 out of 100 for participants aged 76–80.

### Think-aloud data and post-task interviews

This section presents the results of the thematic analysis that was carried out on the think aloud data and post-task interview data collected during the user trial sessions.

#### Performance expectancy (PE)

All fifteen participants suggested at least one practical use for the game. The majority of participants (twelve) believed the game could be useful for everyone, as a reminder of safe practice within the home. Even those who believed the game was overly simplistic and already felt aware of the hazards present in the game believed it could be beneficial to be reminded of these hazards via the game.

*“If you go through it and sail through and think ‘well, that’s a doddle’ then you know you’re very aware, but if you don’t then you think. It might make somebody think twice*.” (Laura).

However, one participant pointed out that while users may see and understand the game, it may not affect their behaviour.

*“I think everyone my age should see something like this. In a way, it’s like a safety advertisement. Mind you, there are ads for smoking… but it doesn’t always work.” (Daniel).*

One participant specified that the reminder may encourage people to return home and make behavioural changes;

*“I suppose it makes you feel that you can go around your own home and see if there are things that are similar to that they’ve had on screen, that you could put up or remove.” (Charlotte).*

Five of the twelve felt that it was important *“particularly for the elderly”* (Matthew), and especially to enable older adults to remain living in their homes and not enter care or hospital, for emotional and financial reasons.

*“You’re really highlighting the type of hazards that they have in their rooms, which is so important for them [older adults] to stay at home, especially these days when people want to stay in their home. They can’t afford to go into a home.” (Karen).*

#### Effort expectancy (EE): the degree of ease associated with the use of the system

Most participants described the game as a positive learning experience despite difficulties with instructions and controls. Eight of the participants commented on the instructions provided in the game, with regard to both clarity and legibility of instructions.

*“The initial instructions were nearly incomprehensible because so much information was pushed at you so quickly before you got the hang of the, how the system worked.”* (Ethan).

*“To be truthful I didn’t read all of it, I thought,” “well, let’s just go with it and see what happens.”* (Joshua).

Eight participants commented how difficult the game was to play, in terms of physically operating the system. Depending on what technology the participant was used to using, either the mouse or the uses of the keyboard were challenging. Using the keyboard in combination with the mouse was also a challenge.

“*I’m finding it very difficult to use this mouse to go round.*” (Kate).

“I *forgot about these arrows, I should have used them before, shouldn’t* I?” (Thomas).

“*The business about ‘W, A, S, and D’ [control keys] - why not use the direction keys*?” (Daniel).

“*This [navigating] I’m finding tricky. I’m not used to doing this at all [using keyboard]. I’m just used to doing everything with the mouse, so the fact you have to join the two.”* (Laura).

One participant suggested that the game controls were overly-complicated and that the game would be better on an alternative platform such as on a tablet.

*“I didn’t find the navigation very easy, it was very crude… It could be made into an app that you could do on the iPad, then you’d get even older people because I think older people are better at using iPads than they are at computers.”* (Deborah).

With regard to the difficulty of the content (i.e. finding hazards) five participants believed the hazards were *“pitched at the right level”* (Daniel). One criticism was that the game became “*repetitive*” (Deborah).

Four participants reported that they were not always aware of hazards, and were simply clicking on all available items until a hazard was correct.

“*I was just fishing really, I’m not clever enough*.” (Matthew).

#### Social influence (SI): the degree to which an individual perceives that important others believe he or she should use the new system

Participants primarily believed the game would be useful if targeted to specific populations. One participant thought it was not useful for her but may be her aging parents.

*“… I wouldn’t say it’s got too much relevance for me because I’m only mid-fifties and I’m still pretty able but thinking of my in-laws who are now both over eighty…you know, people like them yes, I could definitely see it being useful”* (Deborah).

Four participants felt that while they were aware of the messages contained in the game, they felt others – especially informal carers and children – would benefit from playing in order to be more aware and considerate when visiting the homes of elderly relatives.

*“From school children at kindergarten. It’s a very, very good thing to get young people in on things like that, and they can tell their parents, “Daddy, you left this out”.” (Thomas).*

Daniel suggested the game could be displayed as a still image on screens in GP surgeries. Notably, one participant critiqued the game, asking *“is it a game?” (Matthew). The participant believed the game was not competitive or exciting enough to be considered a game, and should be a “training tool”:*

“*I don’t know anything about games because I don’t play them but um, a game is something where you have a little battle with things I suppose*.” (Matthew).

Participants generally used “you” or “they or them” when describing scenarios in which a game hazard could cause a fall for example: *“The people who live in this house do some very strange things”* (Kesia)*.* Some participants also ascribed behavioral traits to the virtual game environment home owners *“Oh, oh dear no you wouldn’t leave an iron on the floor. Well I suppose you would if you were stupid”* (Ethan). Some of the participants were not able to identify falls hazards to self with hazards that were associated with “others” older and frailer than themselves.

“*You have got a lot of elderly people, this is my experience I’m afraid, and some of whom are, a bit thoughtless… and they do tend to leave stuff around. And they do tend not to always look where they’re going. And it might be a good jog for them.*” (Ethan).

### Additional falls hazards identified by participants

Many of the participants identified items that they felt were hazards, however, these were not identified as hazards within the game, i.e. no score was awarded for clicking on these items, although participants felt that they should have been awarded points for identifying these. Table 4 presents all of the items that participants proposed should be identified as hazards, along with quotes from respective participants justifying why they believed these items to be fall hazards.

**Table 4:** Additional items deemed as hazards by participants

Name	Hazard	Quote
Lucy	Mats	*Mats I think are ambiguous, because unless they are completely fixed to the floor, you know, they’re trip hazards.*
Daniel	Layout of furniture	*The edge of the desk is just beyond the door. So you walk in and hit… for furniture you want rounded corners.*
Thomas	Layout of furniture	*That furniture is partly obstructing the door way… someone who has designed the game hasn’t thought about that.*
Matthew	Doors	*The [bathroom] door opens that way, does it? So it opens… it could hit you in the back or something.*
Matthew	Swivel office chair	*Oh, that’s a funny object: that chair. They swivel and if you hold on to it and it swivels… if you’re not too steady on your pins, it could potentially throw you off balance.*
Joshua	Positioning of ‘safe’ bath rail	*I would’ve suggested it [the bath rail] should be lower. You’re never going to reach that in a sitting position.*
Thomas	Positioning of ‘safe’ bath rail	*Oh, very high that rail, isn’t it!*
Joshua	Positioning of ‘safe’ toilet roll	*I don’t think the toilet is necessarily a fall hazard, but certainly where the toilet roll is… reaching.*
Lucy	Table in Lounge	*This table looks uneven to me, because it seems to be balanced on something… is that a trip hazard? It could lead to a fall.*
Thomas	Cooking oil by stove	*This object [oil] is in a safe place? But it’s right next to the cooker!*
Daniel	Cooking oil by stove	*Would you put it [oil] there… I don’t think so. You get flames coming out of the saucepan, and heat.*
Thomas	Lack of pull cord in bathroom	*Yeah, but I’ll tell you also, inside a bathroom, you shouldn’t have a finger switch.*
Charlotte	Lack of pull cord in bathroom	*But you’re not allowed to have a light inside the bathroom, I know that because we build our own ourselves.*
Deborah	Wooden Stairs	*Your stairs did look as though they were just wood and that could be, you know, you might slip on stairs - it might be better if they have a carpet.*
Karen	Balusters on stairs too far apart	*Somebody could fall through the stairs - there’s quite a few - the gaps in the stairs are quite wide, really, aren’t they?*

### Telephone interviews: impact of playing the game

Ten of fifteen participants reported discussing fall risks from the game with friends and family afterward. There was evidence of increased safety awareness and a fear that someone else may be hurt as a result of a falls hazard.

*“When I say "I've made no changes", I do feel more aware of potential hazards, and so whilst not actually affecting any changes I take care not to allow any to be around. I try not to create any, let's put it that way.” (Matthew)*

Another participant reported that although he treated his new awareness of falls hazards like a joke, it has in reality had some useful impact on his awareness and his behaviour.

*“I keep saying “trip hazard” and things like that. Primarily on the stairs and shoes lying around. We treat it like a joke but I guess they are there and it does actually bring it to the forefront of your mind.” (Joshua).*

Most of the participants wanted to share their experience. Three participants reported that they have identified hazards in other people’s homes and have spoken to them about being more safety-conscious.

*‘I discussed it with my husband, but as a family we were very aware anyway. At this moment in time I don’t have any elderly people who need that information. I have discussed it with lots of people but that’s in general terms’ (laura).*

In addition, there was evidence of changes made to the home environment. This ranged from being more aware of cables and toys on the floor (Kate), from arranging shelves to be lowered (Emily), rethinking the design of the bathroom when it is modified to have rails included (Daniel).

Three participants reported that they have identified hazards in other people’s homes and have spoken to them about being more safety-conscious. The interventions included speaking to a neighbour about slippery mats (Deborah), lifting up loose rugs in her mother’s house (Keisa, Daniel). Some of the participants mentioned fall hazard’s which they perceived were missing from the game. These were related to both intrinsic and extrinsic factors. The most frequently remembered fall hazards were lighting and general fall clutter. Table 5 presents the fall hazards that were recalled by participants and the associated frequency of recall for each respective item.

**Table 5:** Fall hazards recalled after 3 weeks and associated frequencies of recall

Fall Hazards Recalled (3 weeks post gameplay)	Frequency of Hazard recalled by participants
Lighting	7
General Floor Clutter	6
Flex (across doorway)	4
Rug/Mat	4
Staircase Hazards	4
Reaching (things at height)	3
Bathroom Grab Rail	2
Water Puddle	2

## Discussion

This is the first study to examine a serious game for falls prevention education relating to extrinsic risk factors. The findings from our study suggest that education gaming needs to be considered as an additional tool to educate community adults about falls within the home. Our research supports the use of gaming to change behaviour within healthy adults living in the community. In addition existing research supports the notion that education on the use of environmental cues may reduce falls risks [38], and that the delivery of training programmes that focus on environmental falls risks can reduce the need for emergency falls risk interventions [39]. Primary health promotion is essential to avoid associated economic and quality of life issues associated with falls [40]. Currently NICE guidance only recommend primary health promotion approaches such as home hazard detection and education as part of a multifactorial intervention, due to limited evidence [16]. Early education prior to a fall is an essential part of active ageing approach and or as one component of future proofing homes for ageing [41]. It is essential to reduce falls since 30–50% of current falls are related to the physical environment [5]. Fall hazards that were most memorable were either those most common (floor clutter) or those most challenging to participants playing the game (lighting). Lighting is of particular importance since secondary data analysis of WHO review of data collected across eight European cities found that inadequate light in housing is independently associated with depression and falls [42]. This is consistent with what is known as the “bizarreness effect” [43], whereby unusual elements (the experiment compared recall of usual and unusual sentences), or items that warrant discussion, made the most impact and were most likely to be recalled. Likewise, in a study of word recall [44] found that negative emotional words were better recalled than neutral words. In addition, there is some evidence that content delivered by virtual trainers may be capable of delivering more memorable educational content, compared with content that is delivered by human trainers [45].

The World Health Organisation [5] suggests it is important to highlight falls risks amongst the whole community. There is evidence that our falls education game could be used to achieve this aim. Our research has identified a tendency for adults to perceive falls as a phenomenon that affects other people, and not only as a risk to the self. In particular, falls were associated with ‘older adults’ and not viewed as an issue that could only impact upon them. This is consistent with previous work by [46] who found that a large majority of older adults do not adhere to falls prevention recommendations and by [47, 48] who found that older people did not accept falls prevention advise as they viewed it as a potential threat to their identity and autonomy. Factors that have been cited as influencing the success of education interventions is whether people find it personally relevant [49, 50]. However, in our study the participants expressed their autonomy by adopting a ‘societal’ approach in that they became aware of the prevention of falls in others and the game as a ‘safety advert’. Therefore, reducing falls risk from a caregiver perspective rather than a personal one may be a more realistic aim for future effective serious gaming in this context. Indeed including the education of caregivers and family members on falls risk factors has been found to be an important part of delivering effective falls prevention education [51].

Understanding the attitudes of the end-user is important, as the success of a technology depends not on whether or not it is effective when used, but whether or not it will be used in actuality [35, 52]. The game on average took 18 min to complete. We did not find any comparable reading times for a falls leaflet. Whilst the users rated the game on usability above average it was noted that SUS scores increased with age it is important that researchers remain aware of errors in satisfaction ratings resulting from cultural differences of users [53]. In this instance, it is age. However, we have found that researchers have neglected to research the relationship between SUS scores and age. Bangor et al. [37] have raised this point. Vaziri et al. [54] study contradicts our findings. In this study the younger participants rated the falls system more usable than the older participants (Over 72 years). This is a small sample though, so we recommend that further research is needed to explore whether this was an anomaly or whether there is further knowledge to be gleaned from this. Nevertheless, this is a particularly interesting finding and further research is required to establish whether indeed this falls game is more usable the older the user is, or whether this finding may be explained by some other moderating or mediating factor. Existing research has found that there are a number of moderating factors that affect older adults’ motivation towards using falls prevention technologies, usability being one of the key factors that impact on use [55].

There are numerous future research activities that have arisen from the results of this study. Although this study has provided in-depth insights into the experiences of older adults playing the Falls Sensei game, there is a need for further iterations of design, development and user testing. Future research should include a larger scale randomised control trial to establish the extent to which playing serious games such as Falls Sensei enables individuals to learn about falls hazards compared with the effectiveness of traditional forms of falls education, such as the use of information leaflets. Furthermore, some of our results indicate that Falls Sensei may be a game that is applicable to other cohorts, such as children, young adults, and carers. Indeed, there are significant benefits to acquiring knowledge about environmental falls hazards across the full range of the population [5]. Therefore, there is a need to carry out further research with such cohorts to establish whether this is the case, and to identify whether/how the game design and functionality should be adapted to maximise effectiveness with a variety of user groups. There is also a need to further explore the extent to which users are willing to engage with and play the game in outside of the controlled experimental setting. The current findings indicate that participants appear to be indifferent about the prospect of playing the game regularly (SUS item S1). If such a game is to have significant impact on the wider population, it is important that users feel motivated to engage with and play the game in the first instance. Further research is required to explore how the Falls Sensei game can be adapted to increase users’ motivation to engage and return to play the game frequently. Increasing the level of difficulty in-line with player performance and widening the variety of falls hazards, may be important avenues to explore to enhance and maintain player engagement [56]. Indeed, presenting challenges that are in-step with player ability is seen as an especially strong predictor of continued engagement and is a key predictor of engagement in digital educational games [57]. Furthermore, there may be value in exploring adaptations of the game narrative to take into account that there is existing evidence that older adults do not engage with falls prevention interventions if they are not seen as being compatible with perceptions of positive identity, or as being personally relevant [47, 48]. As a result of the users’ critique of the game, it is evident that the game instructions and progress updates require further review and refinement. Readability of instructions are a common issue with education leaflets. There is a need to consider alternative hardware options (phone, tablet, swipe screens) especially disability friendly set-ups (e.g. for arthritic hands or low vision) and enhance game interactivity to improve interaction scenarios (e.g. phone ringing). The issues identified are consistent with work by Lee and Kozar [58] and Bhatia et al. [59] who both suggest that legibility is a key aspect of usability.

Therefore, there are a number of recommendations for future work that emerge from the findings of this research

- Recommendation 1) There is a need for more research exploring the effectiveness of serious games such as Falls Sensei compared with more traditional forms of falls education
- Recommendation 2) Further research is required to explore the link between serious 3D exploration games and how usability evaluations of these games vary with age
- Recommendation 3) There is a need to explore the effectiveness of such games, in terms of educational value, for a range of different cohorts including carers, children and young adults
- Recommendation 4) Explore how such games can be designed to increase users’ motivation to engage and return to playing the game frequently
- Recommendation 5) Explore how effective such games are on a range of hardware platforms including mobile phones, tablet computers and swipe screens.

## Conclusion

This research offers a promising exploration into using serious games to address extrinsic factors in fall risk reduction. A multi-method triangulation of analysis suggests awareness of home hazard detection was raised by the game, but further research would be needed to draw comparisons with established interventions. Our research has found that serious games can offer an engaging way to learn about home falls risks and has the potential to make an important contribution towards active aging. However, it is important to note that the findings relating to engagement have emerged from participants that were explicitly issued with the task of engaging with, and playing the game as part of the trial, and did not emerge from an audience that elected to play the Falls Sensei game because they believed it would be engaging. Further research is required to explore whether the prospect of playing a falls game such as Falls Sensei is perceived as a potentially more engaging prospect than engaging with more traditional forms of falls prevention education tasks/activities. What was of interest is that older adults, although may not be willing to make immediate changes, were advocates of home falls safety to prevent harm in others. There was evidence that as a consequence of playing the game, some older adults became more aware for the need to adapt their own homes in the future.

## Abbreviations

EE: Effort Expectancy
PE: Performance Expectancy
SI: Social Influence
SUS: Systems Usability Scale
UTAUT: Unified Theory of Acceptance of Use of Technology
WHO: World Health Organisation
